# Use of Mobile and Computer Devices to Support Recovery in People With Serious Mental Illness: Survey Study

**DOI:** 10.2196/12255

**Published:** 2019-02-20

**Authors:** Valerie A Noel, Stephanie C Acquilano, Elizabeth Carpenter-Song, Robert E Drake

**Affiliations:** 1 Westat, Inc Lebanon, NH United States; 2 The Dartmouth Institute for Health Policy and Clinical Practice Geisel School of Medicine at Dartmouth Dartmouth College Lebanon, NH United States; 3 Department of Anthropology Dartmouth College Hanover, NH United States

**Keywords:** app, mental health, mHealth, mobile phone, serious mental illness

## Abstract

**Background:**

Mental health recovery refers to an individual’s experience of gaining a sense of personal control, striving towards one’s life goals, and meeting one’s needs. Although people with serious mental illness own and use electronic devices for general purposes, knowledge of their current use and interest in future use for supporting mental health recovery remains limited.

**Objective:**

This study aimed to identify smartphone, tablet, and computer apps that mental health service recipients use and want to use to support their recovery.

**Methods:**

In this pilot study, we surveyed a convenience sample of 63 mental health service recipients with serious mental illness. The survey assessed current use and interest in mobile and computer devices to support recovery.

**Results:**

Listening to music (60%), accessing the internet (59%), calling (59%), and texting (54%) people were the top functions currently used by participants on their device to support their recovery. Participants expressed interest in learning how to use apps for anxiety/stress management (45%), mood management (45%), monitoring mental health symptoms (43%), cognitive behavioral therapy (40%), sleep (38%), and dialectical behavior therapy (38%) to support their recovery.

**Conclusions:**

Mental health service recipients currently use general functions such as listening to music and calling friends to support recovery. Nevertheless, they reported interest in trying more specific illness-management apps.

## Introduction

The number of smartphones owned by people with serious mental illness (81%) has been approaching the rate of ownership in the general population recently (91%) [1,2], and the use of these devices by people with serious mental illness does not seem to differ substantially from that of the general population [3]. A survey of 457 people with serious mental illness found that a large majority have access to the internet via computers (89%), smartphones (54%), and tablets (32%) [4], which affords communication, socialization, and the opportunity to obtain information. Qualitative research has suggested that people with serious mental illness are interested in, and some may already be using, electronic devices without clinical team involvement to support their recovery [5]. For example, some people with serious mental illness reported using the internet to understand the precautions and side effects of medications; Instagram, to follow people who post daily positive messages; and YouTube, to watch videos that provide guided progressive muscle relaxation [5].

The concept of recovery began among mental health service users, but mental health personnel, including researchers, have adopted the term [6]. Although recovery is an idiosyncratic concept, qualitative research has identified common themes: personal control over illness management, striving towards one’s goals, meeting one’s needs, and having a sense of responsibility [7-9]. People use routines and activities, such as employment and education, and engage with social support systems to promote their recovery [10]. Currently available technology may further empower people to manage their own recovery.

People with serious mental illness have recognized that technology could become a larger part of their recovery in the next few years [4,11]. Researchers are exploring the integration of smartphone and computer-based apps into psychiatric care for medication management, symptom monitoring, and shared decision making [12-14]. However, not all integrations have been adopted successfully by clients and mental health practitioners. Our study examined current use and interest in future use of specific features and apps of electronic devices by people with serious mental illness to support their recovery.

## Methods

### Participants

This study included a convenience sample of people with serious mental illness who were receiving mental health services from one small and one large mental health agency in New Hampshire. Of the 68 people who completed the survey, we excluded five participants from data analysis: two participants had inconsistent responses and three participants did not own an electronic device. The final sample consisted of 63 participants (31 men and 32 women). Participants ranged in age from 19 to 75 years (mean 41.6; median 42; SD 13.3), and the majority were white (84%, n=53) and never married (63%, n=40). The highest level of education attained by participants was some college or technical school education (38%, n=24), followed by a high school diploma or equivalent (30%, n=19). Nearly two-thirds of the participants were unemployed and not attending school (60%, n=38).

### Measures

We developed a survey (Multimedia Appendix 1) based on findings from interviews of mental health service recipients [5]. This survey first assessed electronic device ownership and frequency of use. For those who owned a computer, tablet, or smartphone, we asked questions differentiating between general everyday use of these electronic devices and specific use for supporting recovery. The questions addressed several topic areas: frequency of use for general purposes, frequency of use for supporting recovery, ease of use, use of technology within mental health care, interest in trying new technologies, and interest in agency-based technical support services. We know from the qualitative study, which informed the development of this survey, that mental health service recipients use general/nonhealth apps (eg, Instagram, Facebook, and online games) to help them with recovery.

The participants rated whether their clinician discussed technology for supporting recovery, on a scale from 1 (no, never) to 10 (yes, at every visit). Participants also rated how comfortable they felt seeking/searching for help in using their electronic devices, on a scale from 1 (very uncomfortable) to 10 (very comfortable).

Because recovery is a highly individual experience [6], we allowed people to use their own understanding of the recovery process rather than an explicit definition. We pretested the survey for clarity and understanding with two consumers and revised the survey based on their feedback.

### Procedure

The Dartmouth College Committee for Protection of Human Subjects in Hanover, New Hampshire, approved this study, which followed the principles outlined in the Helsinki Declaration. Over 4 weeks, we recruited participants from a community mental health center and a dual diagnosis treatment program in New Hampshire. The community mental health center, located in a rural area, serves approximately 1500 adults with serious mental illness each year. The city-based dual diagnosis treatment program serves between 30 and 40 men with co-occurring serious mental illness and substance use. A researcher and a research assistant approached clients in the waiting room and common areas of these centers, explained the study, and asked whether they would be interested in participating. In a few instances, the case managers approached the clients. Only clients who could provide informed consent and were receiving services at one of the two sites were eligible. We provided eligible, interested clients a tablet or paper-based survey, which included a description of the study and a consent statement to complete the survey. Consenting participants completed the survey within 5 minutes. The researcher and research assistant were available to answer any questions and help those who requested assistance in completing the survey.

### Data Analysis

We identified the five most frequently and least frequently used functions/apps for general everyday purposes. We then identified the five most frequently used functions/apps for supporting recovery. We also identified the top five functions/apps that participants were most interested in using to support their recovery in the future. We then used the Fischer exact test to identify whether these frequencies differed between the two sites. We used the Spearman rank correlation to assess whether age was associated with (1) the extent to which clinicians discuss technology with their clients and (2) the client’s level of comfort with seeking help for using electronic devices.

## Results

### Ownership and Usage

Approximately 90% of the people approached agreed to participate, but we do not have the exact number. The participants owned Android phones (67%, n=42), laptops/computers (63%, n=40), tablets (38%, n=24), and iPhones (29%, n=18). The most frequently used device by participants was Android phones (63%, n=40).

### General Everyday Use

Participants regularly (ie, few times a week or every day) used their devices to access the internet (84%, n=53), make calls (79%, n=50), text (79%, n=50), keep track of time (67%, n=42), access social media (60%, n=38), and track the weather (60%, n=38).

Participants rarely used their devices for mental health symptom monitoring (14%, n=9), sleep (14%, n=9), meditation (14%, n=9), dialectical behavior therapy (13%, n=8), physical symptom monitoring (eg, blood pressure and insulin level; 13%, n=8), or cognitive behavioral therapy (10%, n=6).

### Use to Support Recovery

To specifically support their recovery, participants most commonly listened to music (60%, n=38), accessed the internet (59%, n=37), called (59%, n=37), texted (54%, n=34), and used the clock feature to track time (41%, n=26). Participants’ frequency of use of the apps for either general every day or recovery purposes did not significantly differ between the two sites.

### Interest in Incorporating Technology Into Mental Health Recovery

Participants averaged a score of 3.2 (SD 2.6) on a scale from 1 (no, never) to 10 (yes, at every visit) when describing how frequently their case manager or clinician discussed the ways technology can support their recovery. The frequency of discussing technology with case managers or clinicians did not vary with age (*r*_*s*_=–.19, *P*=.22). Among the two-thirds of participants (67%, n=42) who indicated that they would “probably” or “definitely” try new apps or technology to support their recovery, the most popular areas of interest included anxiety (45%, n=19), mood management (45%, n=19), mental health symptom monitoring (43%, n=18), cognitive behavioral therapy (40%, n=17), sleep (38%, n=16), and dialectical behavior therapy (38%, n=16).

A total of 48% (n=30) of participants found it easy to use new technologies; 29% (n=18) reported that it was sometimes easy and sometimes difficult to use new technologies. Participants reported that they either searched online or solicited help from family or friends when they need help using their device (75%, n=47). They rated their level of comfort with these approaches as 7.6 (SD 3.0) on a scale from 1 (very uncomfortable) to 10 (very comfortable). The level of comfort in seeking support did not vary with age (*r*_*s*_=–.17, *P*=.27). Further, 60% of the participants indicated that they would “definitely” or “probably” work with an agency staff member who could help them use their devices, if such a person were available.

## Discussion

### General Findings

Nearly all participants had access to devices that could connect to the internet. Between 40% and 60% identified specific features/apps they were currently using to support their recovery, namely, listening to music, accessing the internet, calling, texting, and keeping track of time. Two-thirds of the participants indicated that they were interested in trying new technologies to support their recovery. Participants were most interested in learning how to use apps that addressed anxiety, mood management, mental health symptom monitoring, cognitive behavioral therapy, sleep, and dialectical behavior therapy. Participants were moderately comfortable searching the internet or asking family or friends when they needed assistance using their device but were open to using technical support services if they were made available at the mental health center.

### Supporting Recovery

The majority of participants routinely used nonmental health features/apps, specifically those built automatically into electronic devices (eg, internet browser, texting apps, calling apps, and time tracking apps) to support their recovery. For example, one participant used the alarm on his phone for medication reminders, while another used the internet browser to learn more about mental health diagnoses [5]. In the present study, a substantial number of participants were interested in using mental health apps. These apps are publicly available; therefore, the following question arises: Why were the participants not using these apps? First, participants with low income have budget constraints that limit the brands of devices they can own and data plans they can afford, which directly impacts access to electronic resources [5]. Second, clients may view these apps as clinical tools that require support from a clinician. Our study participants reported minimal discussion with their clinicians about using technology to support recovery. Third, clients may not know where to find specific apps or how to decide on which ones to use. Mental health centers have a clear opportunity to involve a staff member with expertise in the field of mental health apps, such as a technology specialist, who can inform both clients and clinicians of vetted tools that may help support recovery efforts [5,15]. Evidence suggests that low-level support from professionals and the involvement of peers in a technology-supporting role would be helpful [16]. The majority of participants in this study were open to using such types of resources.

Between 38% and 45% of participants endorsed interest in apps in six target areas related to mental health, indicating that more than half of the participants are not interested in these apps. Consistent with the principle of shared decision making, researchers and clinicians could begin by taking advantage of the apps people are already using. Based on the study findings, participants found the apps that connect them to others or provide information most helpful for recovery. Clinicians may consider supporting their clients in using these features. Technology specialists may narrow their search to apps that have a social component or provide the latest news in mental health. Researchers developing mental health apps may consider including social networking and components that provide new and changing content about mental health. Researchers and clinicians may also consider social factors that influence the use of electronic devices, such as education and employment.

### Limitations

Our study used a convenience sample in New Hampshire that lacked ethnic/racial diversity. We did not collect information on participants’ diagnoses. Behaviors described here were based on self-report, and people’s self-reported attitudes may not predict their behaviors.

### Conclusions

People with serious mental illness use common features of smartphones, personal computers, and tablets to support their recovery, independent of the care they receive from mental health clinics. Clinicians and researchers may consider including a discussion of the apps clients are already using to monitor how effectively these tools support recovery efforts over time. A large minority of participants expressed interest in mental health-specific apps. Because the combination of interest, support, and acceptance is a key driver of adoption, clinicians and researchers may find successful adoption of these apps by starting with these clients and their choices rather than with all clients and specific apps.

## Appendix

Multimedia Appendix 1 Survey on technology use.
