# Supplementary material for: Use of Mobile and Computer Devices to Support Recovery in People With Serious Mental Illness: Survey Study
Source: JMIR Ment Health. 2019 Feb 20;6(2):e12255. doi: 10.2196/12255 (PMC6401671; doi:10.2196/12255)
Supplement: Multimedia Appendix 1 [file mental_v6i2e12255_app1.pdf]

APPENDIX 1: Technology Use Survey

I. Electronic Devices

Do you own either a Smartphone (e.g., LG, Android, iPhone), Flip phone, Laptop/Computer, or iPad/Tablet?

- o Yes
- o No ***If no, skip to end.***

What type of cell phone do you have?

- o Flip phone ***If flip phone, skip to Section III - Interest in Technology.***
- o Smartphone (e.g., LG, Android, iPhone)
- o I don't have a cell phone but I have a laptop/computer or iPad/tablet

II. Use of Technology

What kinds of electronic devices do you have and how often do you use them?
(Mark only one oval per row)

		Yes:					
	No	every day	few times a week	once a week	once a month	less than once a month	never use it
iPhone	◯	◯	◯	◯	◯	◯	◯
Android	◯	◯	◯	◯	◯	◯	◯
Laptop or Computer	◯	◯	◯	◯	◯	◯	◯
iPad or Tablet	◯	◯	◯	◯	◯	◯	◯

How often do you use them for...
(Mark only one oval per row)

	Every day	Few times a week	Once a week	Few times a month	Almost never	Never
Calling	◯	◯	◯	◯	◯	◯
Texting	◯	◯	◯	◯	◯	◯
Music	◯	◯	◯	◯	◯	◯
Social media (e.g., Facebook, Snapchat,Twitter, Instagram)	◯	◯	◯	◯	◯	◯
Internet (e.g., Google, Internet Explorer, Firefox)	◯	◯	◯	◯	◯	◯
Email	◯	◯	◯	◯	◯	◯
Games I play alone	◯	◯	◯	◯	◯	◯
Games I play with others	◯	◯	◯	◯	◯	◯
Reading books	◯	◯	◯	◯	◯	◯
News/current events	◯	◯	◯	◯	◯	◯
Calendar/appointment book	◯	◯	◯	◯	◯	◯
Calculator	◯	◯	◯	◯	◯	◯
Weather	◯	◯	◯	◯	◯	◯
Clock/alarm	◯	◯	◯	◯	◯	◯

Personal assistant (e.g., Siri, Cortana, Google)	○	○	○	○	○	○
Notepad/memos	○	○	○	○	○	○
Food/nutrition	○	○	○	○	○	○
Fitness	○	○	○	○	○	○
Physical symptom monitoring (e.g., blood pressure or insulin level)	○	○	○	○	○	○
Mental health symptom monitoring (e.g., mood or anxiety)	○	○	○	○	○	○
Mood management	○	○	○	○	○	○
Anxiety/stress management	○	○	○	○	○	○
Cognitive Behavioral therapy (CBT)	○	○	○	○	○	○
Dialectical Behavior Therapy (DBT)	○	○	○	○	○	○
Meditation	○	○	○	○	○	○
Relaxation	○	○	○	○	○	○
Sleep	○	○	○	○	○	○

Which features/apps help to support your recovery?
Mark only one oval per row.

	Yes	Yes but would like to learn how to use it more	No	No, but I would like to learn how to use it
Phone	○	○	○	○
Text	○	○	○	○
Music	○	○	○	○
Social media (e.g., Facebook, Snapchat, Twitter, Instagram)	○	○	○	○
Internet (e.g., Google, Internet Explorer, Firefox)	○	○	○	○
Email	○	○	○	○
Maps/navigation	○	○	○	○
Games I play alone	○	○	○	○
Games I play with others	○	○	○	○
Reading books	○	○	○	○
News/current events	○	○	○	○
Calendar/appointment book	○	○	○	○
Calculator	○	○	○	○
Weather	○	○	○	○
Clock/alarm	○	○	○	○
Notepad/memos	○	○	○	○
Food/nutrition	○	○	○	○
Fitness	○	○	○	○
Physical symptom monitoring (e.g., blood pressure or insulin level)	○	○	○	○
Mental health symptom monitoring (e.g., mood or anxiety)	○	○	○	○
Mood management	○	○	○	○
Anxiety/stress management	○	○	○	○
Cognitive Behavioral therapy (CBT)	○	○	○	○
Dialectical Behavior Therapy	○	○	○	○

(DBT)				
Meditation	◯	◯	◯	◯
Relaxation	◯	◯	◯	◯
Sleep	◯	◯	◯	◯

How easy is it to figure out, on your own, how a new app or electronic device works?
Mark only one oval.

- o Very easy
- o Easy
- o Sometimes easy/sometimes hard
- o Hard
- o Very hard

When you don't know how to do something on your device, what are you most likely to do?
Mark only one oval.

- o Nothing
- o Get help from family or friends
- o Ask my clinician/case manager
- o Ask my mobile service provider (e.g., Verizon, Sprint, etc.)
- o Go to the place where I purchased the device (e.g., Best Buy, Walmart, etc.)
- o Research it online (e.g., "Google it")

How comfortable do you feel doing this?
Mark only one oval.

	1	2	3	4	5	6	7	8	9	10	
Very Uncomfortable	◯	◯	◯	◯	◯	◯	◯	◯	◯	◯	Very Comfortable

III. Interest in Technology

Does your care manager or mentor ever talk with you about using technology to support your recovery?
Mark only one oval.

	1	2	3	4	5	6	7	8	9	10	
No, never	◯	◯	◯	◯	◯	◯	◯	◯	◯	◯	Yes, at every visit

What is your interest in trying new technologies?
Mark only one oval.

- o Very interested, would definitely try new apps/technology
- o Somewhat interested, would probably try new apps/technology
- o Unsure, may or may not try new apps/technology
- o Not interested, would probably not try new apps/technology

If there was a staff member here at the clinic who was supposed to help people with their electronic devices, would you to go that staff member for help with your devices?
Mark only one oval.

- o Yes, definitely
- o Probably
- o Maybe/maybe not
- o Probably not
- o No, definitely not
